# Web-Based Forums for People Experiencing Substance Use or Gambling Disorders: Scoping Review

**DOI:** 10.2196/49010

**Published:** 2024-06-17

**Authors:** Annette Peart, Freya Horn, Rachel Petukhova, Anthony Barnett, Dan I Lubman

**Affiliations:** 1 Eastern Health Clinical School Monash University Richmond, Victoria Australia

**Keywords:** web-based forums, peer support, substance use, gambling, scoping, review method, review methodology, forum, forums, substance abuse, addiction, addictive, addictions, peer-based, peer support

## Abstract

**Background:**

For people experiencing substance use or gambling disorders, web-based peer-supported forums are a space where they can share their experiences, gather around a collective goal, and find mutual support. Web-based peer support can help to overcome barriers to attending face-to-face meetings by enabling people experiencing addiction to seek support beyond their physical location and with the benefit of anonymity if desired. Understanding who participates in web-based peer-supported forums (and how), and the principles underpinning forums, can also assist those interested in designing or implementing similar platforms.

**Objective:**

This study aims to review the literature on how people experiencing substance use or gambling disorders, and their family, friends, and supporters, use and participate in web-based peer-supported forums. Specifically, we asked the following research questions: (1) What are the characteristics of people who use web-based peer-supported substance use or gambling-focused forums? (2) How do people participate in web-based peer-supported forums? (3) What are the key principles reportedly underpinning the web-based peer-supported forums? (4) What are the reported outcomes of web-based peer-supported forums?

**Methods:**

Inclusion criteria for our scoping review were peer-reviewed primary studies reporting on web-based addiction forums for adults and available in English. A primary search of 10 databases occurred in June 2021, with 2 subsequent citation searches of included studies in September 2022 and February 2024.

**Results:**

Of the 14 included studies, the majority of web-based peer-supported forums reported were aimed specifically for, or largely used by, people experiencing alcohol problems. Results from the 9 studies that did report demographic data suggest forum users were typically women, aged between 40 years and early 50 years. Participation in web-based peer-supported forums was reported quantitatively and qualitatively. The forums reportedly were underpinned by a range of key principles, mostly mutual help approaches and recovery identity formation. Only 3 included studies reported on outcomes for forum users.

**Conclusions:**

Web-based peer-supported forums are used by people experiencing addiction in a number of ways, to share information and experiences, and give and receive support. Seeking web-based support offers an alternative approach to traditional face-to-face support options, and may reduce some barriers to engaging in peer support.

## Introduction

People with substance use disorders or experiencing gambling problems can feel shame and stigma, contributing to increased social isolation and delayed help-seeking [1-4]. Peer support, which involves the sharing of experiences, knowledge, support, and practical help among people with lived experiences of similar issues [5,6], has a long history in substance use and gambling recovery [7,8]. Peer support has been particularly effective in overcoming shame and stigma, creating spaces built on shared experiences where people can connect safely and learn about help-seeking [5,7].

Peer support has traditionally been accessed through face-to-face meetings, such as 12-step or within therapeutic communities [7]. However, many people face barriers to accessing face-to-face peer support, including geographical distance, regional and rural service gaps, and fear of stigma, as well as insufficient time to travel to and attend meetings, amid the general demands of work, family, and life. The expansion of the digital world means that opportunities to connect with peers have grown. Web-based peer support can help to overcome barriers to attending face-to-face meetings by enabling people experiencing addiction to seek support beyond their physical location and with the benefit of anonymity if desired [9].

Increasingly, web-based peer support is available in web-based discussion communities or forums hosted on social media platforms or websites. Web-based forum users initiate discussions by starting a thread, responding to users’ threads, or scrolling or searching past threads for content of interest. Forums typically only require an email address and self-selected username to post a discussion. Web-based forums, therefore, provide anonymity, access to peer support at any hour and from any location, and the option for both synchronous and asynchronous discussions [10-12].

For a range of health issues, engagement in web-based forums provides benefits for forum users, including improvements in mood, connectedness, and social support, and access to practical support and advice [13-15]. For people experiencing substance use or gambling disorders, web-based forums are a space where they can share their experiences, gather around a collective goal, and find mutual support [12,16]. This type of sharing, particularly with peers who have lived or living experience of substance use or gambling disorders is a key principle of recovery [17].

While there are limited reviews specifically on substance use or gambling web-based forums, a recent systematic review looked at digital recovery support services (D-RSS) led by substance use specialists or peers for people with a substance use disorder [18]. Ashford et al [18] identified 22 studies of various web-based services, including peer-based communities such as recovery social networking sites and web-based forums, and nonpeer-based interventions such as mobile text messaging and digital module-based learning. The authors found that while the evidence of the effectiveness of D-RSS for improving recovery-related outcomes was currently lacking, these services have high use and could overcome accessibility and availability barriers. Ashford et al [18] also called for further exploration of how, why, and to what extent people participate in D-RSS. Given the diversity of services identified in the systematic review, focusing on a subset, such as web-based peer-supported forums, may provide clearer information on the use and utility of these services. Additionally, by including gambling-focused digital services, which were omitted from the review by Ashford et al [18], we can broaden the focus to include web-based peer-supported forums on any addiction. Understanding who participates in the web-based peer-supported forums (and how), and the principles underpinning forums, can also assist those interested in designing or implementing similar platforms.

We conducted a scoping review on how people experiencing substance use or gambling disorders, or their family, friends, and supporters, use and participate in web-based peer-supported forums. Specifically, we asked the following research questions: (1) What are the characteristics of people who use web-based peer-supported substance use or gambling-focused forums? (2) How do people participate in web-based peer-supported forums? (3) What are the key principles reportedly underpinning the web-based peer-supported forums? (4) What are the reported outcomes of web-based peer-supported forums?

## Methods

Scoping reviews can assist in summarizing findings from a heterogeneous body of knowledge and identifying gaps in the literature [19]. Our work was structured around the 5 stages of the Arksey and O’Malley [20] framework and informed by Levac et al’s [21] refinements to this framework.

We used a 2-step search strategy. First, in June 2021, we searched MEDLINE (OVID), PsycINFO, CINAHL, Emcare (OVID), AMED, Web of Science, Scopus, Central Register of Controlled Trials, Informit, and Sociological Abstracts using the terms: addictive behavior, substance-related disorders, gambling, web-based social networking, and term variations (eg, web-based, internet, community, dependence, and addiction). The complete search strategy for MEDLINE can be found in Multimedia Appendix 1. An academic librarian reviewed and refined our search strategy. Second, in September 2022 and February 2024, we performed a citation search of our original included studies to obtain more recent studies.

Our inclusion criteria were as follows: (1) peer-reviewed primary studies, (2) reporting on web-based addiction-focused forums, (3) web-based forums designed for adults (aged 18 years and older), and (4) those available in English. In relation to the second inclusion criterion, the studies needed to report results based on data derived from forum posts or metrics. We excluded gray literature and full-text conference proceedings. Studies where reference was made to web-based forums, but data were not derived from the forums, were excluded; for example, studies where data were about participant perceptions of forums in general. We also excluded studies where addiction was not the focus of the forum, or interventions were apps, rather than forums. No restrictions on the date of publication were used.

We used Covidence (Veritas Health Innovation), a web-based collaboration software platform, to facilitate screening study titles and abstracts, and again for full-text review. Two independent authors (AP, RP, FH, or AB) reviewed the titles and abstracts of studies for inclusion or exclusion. Two independent authors (AP, RP, FH, or AB) then extracted the data from the full text of the included studies. We resolved conflicts on study selection by consensus and team discussion if needed.

Using the research questions as a guide, we developed a data extraction template in Covidence. We extracted the following data from the included studies: study characteristics (eg, country of origin, year, and study type); web-based forum characteristics (eg, principles, models or theories reported, forum user characteristics, use, and participation); and outcomes reported. After each author independently charted the data, we discussed the results and updated the form through an iterative process. A formal assessment of methodological quality was not part of this scoping review.

We used Microsoft Word to visually display our data extraction as tables, and for the analysis, we discussed descriptive findings as a group. We grouped the studies according to our research questions and summarized the key findings numerically and thematically. We met regularly throughout the project, incorporating reflexive elements to consider how we analyzed the data and where data best fit to answer our research questions.

## Results

### Selection of Sources

**Figure 1:**
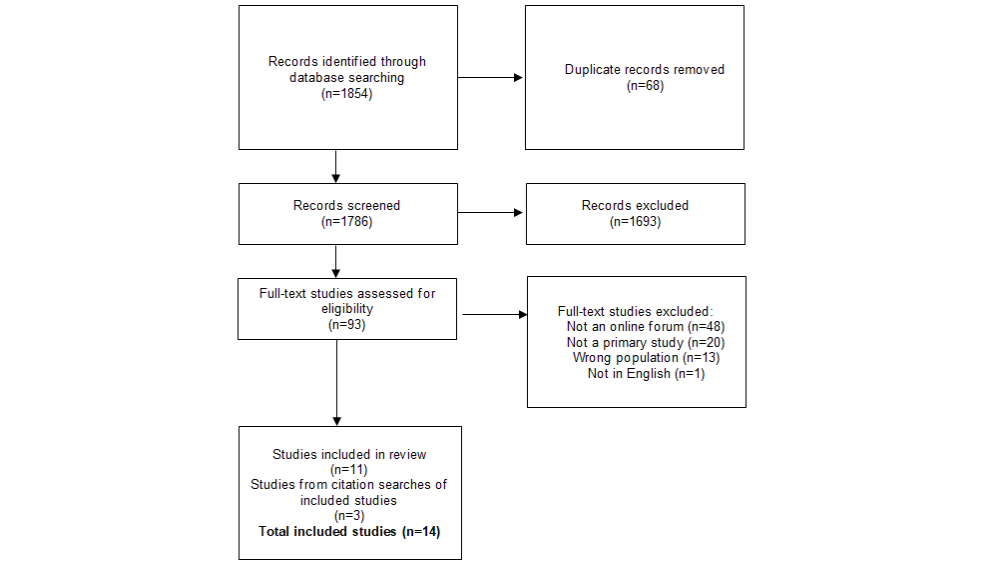
PRISMA (Preferred Reporting Items for Systematic Reviews and Meta-Analyses) flow chart.

Our initial search terms generated 1854 records from the databases. We removed 68 duplicates, leaving 1786 to be screened according to title and abstract, of which 1693 were excluded. After that screening, 93 full-text studies were reviewed, and 82 were excluded, leaving 11 studies included as a result of the database search. The citation search of included studies resulted in the addition of 2 further studies in September 2022, and 1 further study in February 2024. The final number of included studies was 14 (Figure 1).

### Characteristics of Sources

Of the 14 included studies, 5 studies originated in the United Kingdom, 3 studies in each of the United States and Australia, 1 study in each of Canada and Russia, and 1 study not confined to a specific country, reporting on a range of web-based peer-supported forums. The majority of web-based peer-supported forums reported in the studies were aimed specifically for, or largely used by, people experiencing alcohol problems. Two studies were not specific to any substance; however, these reported that more than half of the people using the web-based forum were seeking alcohol support (306/343, 91.6% alcohol [22] and 79/123, 65% alcohol [23]). Two forums focused on people with opioid concerns (1 codeine-specific [24] and 1 for pregnant women with opioid use problems [25]), and 2 focused on gambling concerns [26,27]. Two web-based peer-supported forums were the focus of 5 studies: Hello Sunday Morning ([HSM] Australia, 3 studies) [28-30] and Soberistas (United Kingdom, 2 studies) [12,31]. For 3 studies, the authors did not name the web-based forums they reported on [11,25,27]. Table 1 outlines the characteristics of the included studies.

**Table 1:** Characteristics of included studies.

Authors (date)	Country	Source description of web-based forum	Primary substance or behavior	Reported purpose of forum	Forum moderation	Sample size
Bergman et al (2017) [23]	United States	In The Rooms, a recovery social network site with 430,000 registered users	Substance use	For people in or seeking substance use recovery, including recovery resources and recovery-based discussion boards	Moderated	123 participants
Black et al (2020) [28]	Australia	Hello Sunday Morning (HSM), free website and app with over 40,000 participants	Alcohol	A community for facilitating action and reflection, at individual and collective levels, rather than information provision	Not specified	24 participants
Bradley and James (2021) [26]	United Kingdom	“My Journal” on Gambling Therapy website	Gambling	For people to post about life before, during, and after gambling problems	Moderated	First posts from 2298 threads
Carah et al (2017) [29]	Australia	HSM (refer to Black, et al [28])	Alcohol	As above [28]	Not specified	13,878 blog posts from 7890 registered users
Chambers et al (2017) [31]	United Kingdom	Soberistas, web-based mutual aid group with 1828 paid members and 2000 browsers	Alcohol	For people trying to resolve their problematic drinking patterns	Moderated	31 participants
Colditz et al (2023) [32]	No specific country	r∕StopDrinking, subreddit	Alcohol	For people who are trying to abstain from alcohol use	Moderated	1460 direct responses to posts
Kirkman et al (2018) [30]	Australia	HSM (refer to Black, et al [28])	Alcohol	As above [28]	Not specified	1917 participants
Lee and Cooper (2019) [24]	United Kingdom	Mumsnet social media forum	Codeine	A parenting website with alcohol and other drug sections for people seeking support	Moderated	25 threads comprising 757 individual posts
Liang et al (2021) [25]	United States	Unnamed web-based health community with a long-standing history and active user participation	Opioids during pregnancy	Web-based health community	Not specified	200 posts
Lyytikäinen (2016) [33]	Russia	Alcoholics Anonymous, “newcomers” subsection of an asynchronous web-based forum comprising over 10,000 posts	Alcohol	For people to discuss problems related to drinking and their recovery journeys	Not specified	10 most recent threads, including 617 posts by more than 35 members
Mudry and Strong (2013) [27]	Canada	Unnamed web-based support forum of 3253 members	Gambling	Support for people concerned about problem gambling	Moderated	1791 posts of 11 members
Sanger et al (2019) [11]	United Kingdom and United States	Five unnamed web-based support groups ranging in size	Alcohol	Support groups using non–12-step philosophies	Moderated	25 participants
Schwebel and Orban (2022) [22]	United States	Harm reduction, Abstinence, and Moderation Support (HAMS), a private forum-based and social media support group	Substance use	Originally a support group for people to change their alcohol use, expanded to other substances	Moderated	343 participants
Sinclair et al (2017) [12]	United Kingdom	Soberistas [31]	Alcohol	As above [31]	Moderated	432 participants

### What Are the Characteristics of People Who Use Peer-Based Web-Based Forums?

Reporting of the characteristics of web-based peer-supported forum users in the included studies varied. Due to the use of either anonymous posts or web-scraping for data collection, the authors of 5 of the studies provided no demographic information [24,26,27,29,32]. Results from studies that did report demographic data (n=9 [11,12,22,23,25,28,30,31,33]) suggest forum users were typically women, aged between 40 years and early 50 years (reported median age range 41.5-50.8 years). In the 9 papers that reported on gender [11,12,22,23,25,28,30,31,33], more than half of the sampled forum users were women: this ranged from 57% women [23] to 94% women [12]. In studies where information on education or socioeconomic status was reported (n=3 [11,12,28]), most forum users held higher education qualifications or were classified as being of moderate to high socioeconomic status. In 3 studies, the authors reported on race [11,22,23], and the majority of participants were reported to be White (>90%).

Current substance use of sampled web-based peer-supported forum users was reported in 6 studies [12,23,25,28,30,31]; however, the information provided varied. Several authors reported measures that indicated high levels of current use [25,28,30]. For example, in 2 studies reporting on the HSM forum, 42% [28] and 54% [30] of forum users’ Alcohol Use Disorders Identification Test [34] responses indicated high-risk or dependent alcohol use at the time of data collection. Similarly, Liang et al [25] reported that 75% of forum users met one or more criteria for an opioid use disorder.

In 3 studies [12,23,31], the authors did not report the severity of substance use problems of web-based peer-supported forum users, instead providing abstinence-related data. The proportion of abstinent and nonabstinent forum users across these 3 papers varied widely. According to Sinclair et al [12], 53% of forum users were currently drinking alcohol or had consumed alcohol in the last month and 18% were abstinent for a year or more. Chambers et al [31] reported that only 23% were current or recent (<1 month) alcohol consumers and 39% were abstinent for a year or more. Bergman et al [23] reported that the majority of forum users were abstinent for a year or more (65%), and only 13% were current or recent alcohol consumers. The forum featured in Bergman et al [23], “InTheRooms” (ITR) comprised a particularly large proportion of long-term abstinent forum users; 21% were abstinent for 5-9 years and 26% were abstinent for 10 or more years. The average duration of continuous abstinence for forum users in the study by Bergman et al [23] was 7.3 (SD 9.3) years.

### How Do People Participate in Peer-Based Web-Based Forums?

Participation in the web-based peer-supported forums, in terms of patterns of use and information sought, varied between the 14 studies. Some authors described forum participation quantitatively such as the number of users (n=7 [12,25-27,29,30,33]), number of posts (n=7 [24-27,29,32,33]), length of time per visit (n=3 [22,23,30]), and frequency of use (n=2 [22,23]).

In 11 studies, how web-based peer-supported forum users participated was also reported qualitatively. These qualitative features are presented in Table 2. Web-based forum users participated in a range of ways, by sharing information and personal experiences, and seeking support (Table 2).

**Table 2:** Qualitative data reported in the included studies.

Authors (year)	Name of the web-based forum	Data analysis methods reported	Qualitative data reported
Black et al (2020) [28]	Hello Sunday Morning (HSM; alcohol)	Thematic analysis of semistructured interviews	HSM attracted people actively seeking help and people not yet seeking help. Forum users viewed HSM in a positive, nonthreatening manner and liked the anonymity and convenience of the mobile format. They joined out of curiosity or desire for a challenge. Forum users liked the support and normalization of experiences through the blogging feature. They were motivated by the goal-setting and self-monitoring components enabled by challenges and weekly check-ins.
Carah et al (2017) [29]	HSM	Text analysis of blog posts, grouping expressions together as related concepts	Forum users’ expressions changed over time. In the first month, they set goals, and described current drinking practices, hopes and anxieties, and early efforts to change. After the first month, forum users reported on change efforts and challenges, and reflected on their place in a drinking culture. They evaluated their efforts to change and presented “findings” and “theorised” them to advise others.
Chambers et al (2017) [31]	Soberistas (alcohol)	Grounded theory techniques to analyze in-depth interviews	Key stages of engagement, through which forum users’ identities were constructed and adjusted to support recovery. The most linear and commonly discussed engagement involved transitions through “lurking,” “participating,” “leading,” then “moving on”; coinciding with forum users’ journey from problematic use to “secure in sobriety.”
Colditz et al (2023) [32]	r∕StopDrinking (alcohol)	Constant comparative method	Emotional support included expressions of encouragement, emotional alignment, sympathy, or empathy, in relation to an original post, sobriety-related accomplishment, challenge, or some other quality of the narrative. Appraisal support normalized the original poster’s experiences, negatively appraised drinking behavior, and positively appraised recovery behaviors and outcomes. Informational support included fact-based information, informed opinions, and instructions.
Lee and Cooper (2019) [24]	Mumsnet (codeine)	Thematic analysis of threads	Forum users created posts to request help in relation to usually, but occasionally their relative’s, problems with codeine use and self-reported addiction. Positive and negative descriptions of side effects, problems experiencing withdrawal, and failed attempts to discontinue use were reported. Advice was provided about formal health services or informal approaches, and often anecdotal advice about how to taper or use cold turkey techniques. Arguments and challenges to advice were not uncommon. Shame and stigma were often associated with posts and forum users often wanted to keep codeine use hidden in their lives.
Liang et al (2021) [25]	Unnamed (opioids during pregnancy)	Thematic analysis of posts	The following six themes highlighted self-management support needs: (1) clarity on the impact on pregnancy, (2) clinically validated information on how to reduce dosage, (3) guidelines on safe pain management during pregnancy, (4) information on local child protection procedures, (5) strategies for obtaining support from offline systems, and (6) emotional support for those experiencing negative emotions.
Lyytikäinen (2016) [33]	AA^a^	Content analysis of posts	Forum users gave each other mutual support in going through phases of change. Many started to adopt the philosophy of AA, model the AA life story, and acquire new self-understanding of a sick person with a chronic disease. By engaging, forum users acquired a sense of agency, and being in charge of their lives. The forum created a web-based space where users collectively acted according to AA values, which supported them to do so offline as well.
Mudry and Strong (2013) [27]	Unnamed free support forum (gambling)	Discourse analysis of posts	The following six common discourses were used in the forum: (1) shame and guilt, (2) causality, (3) nature of gambling, (4) gambling as an addiction or illness, (5) control and responsibility, and (6) recovery as a process.
Sanger et al (2019) [11]	Five unnamed web-based support groups (alcohol)	Thematic analysis of semistructured interviews	Most important benefit of groups was finding “someone like me.” Forums provided support without requiring users to follow a set program for recovery. Forum users respected others’ rights to choose their goal for sobriety and how they achieved it.
Sinclair et al (2017) [12]	Soberistas (alcohol)	Coding and summarizing of free-text survey responses	Anonymity, ability to be honest, source of trusted information, and ongoing support were reasons for continued membership.

^a^AA: Alcoholics Anonymous.

Across the 11 studies reporting on these qualitative features of participation, web-based peer-supported forums were a source of information and advice for people in similar circumstances. For example, Liang et al [25] reported that forum users were seeking information on opioid use during pregnancy and advice on self-managing their use. Forum users obtained information about the adverse effects of opioid use during pregnancy, self-managed withdrawal, continued safe use, and child protection and health systems. Similarly, Sinclair et al [12] examined the Soberistas forum where users shared information on alcohol, health, and well-being. Information sharing was reported as useful for all forum users, even passive ones. Chambers et al [31] found “lurking” behavior was common in the Soberistas forum and reported that users who “lurk” without posting were still able to gain important information. However, web-based peer-supported forums could also contain potentially inaccurate advice or misinformation. For example, Lee and Cooper [24] reported that when one forum user suggested using cannabis to help with codeine addiction, this comment was swiftly negated by another user who posted, “be careful with rubbish advice.” While 9 of the 14 papers included forums with moderators, it was unclear how active moderators were regarding posts involving misinformation. Moderator duties were described as providing content, feedback, or support [22,26,27] or removing spam posts or posts that violated community guidelines [12,23,26,27,31].

Many web-based peer-supported forum users also sought emotional support. Lyytikäinen observed that Russian Alcoholics Anonymous forum users made posts that described their current situation and asked for support, and other forum users then offered guidance or motivation (eg, “Stay and recover with us”) [33]. In other studies, forum users shared their personal circumstances. For example, from Lee and Cooper’s study: “My partner has been getting lots and lots of codeine in over-the-counter Nurofen Plus over the years by going to different pharmacies” [24]. Mudry and Strong [27] found that senior forum users shared past experiences of recovery to support others, and this sharing also established their seniority, authority, and legitimacy within the group.

### What Are the Key Principles Reportedly Underpinning Peer-Based Web-Based Forums?

In 11 of the 14 included studies, the authors reported key principles underpinning the web-based peer-supported forums (Table 3). Behavior change models appeared to underpin the HSM forums [28-30]. Bergman et al [23] and Schwebel and Orban [22] referred to the Social Identity Model of Recovery, as described by Best et al [35]. Carah et al’s [29] study of a gambling-focused web-based forum referred to the importance of relationships to help control or stop gambling. Web-based peer-supported forums reportedly founded through mutual aid included the Soberistas forum, described as nonprescriptive, nonjudgmental, and nonreligious [12,31] and the Russian Alcoholics Anonymous forum [33]. Liang et al [25] focused on self-management support, and Mudry and Strong [27] referenced learning and support through others in a community of practice model [36] although the name of the web-based forum was not reported in both studies. Finally, data were analyzed in the subreddit StopDrinking reported by Colditz et al [32] according to House’s [37] conceptual model of social support.

**Table 3:** Key principles of web-based peer-supported forums reported in the primary studies.

Name of web-based forum	Authors (date)	Key forum principles or approaches
Alcoholics Anonymous (AA)	Lyytikäinen (2016) [33]	Phase model of therapeutic change embedded within AA movement.
Gambling Therapy: “My Journal”	Bradley and James (2020) [26]	Forum as a beneficial source of support, fostering growth of relationships to help attempts to control or stop gambling.
Harm reduction, Abstinence, and Moderation Support (HAMS)	Schwebel and Orban (2023) [22]	Recovery through exposure to relatable role models, enhancing motivation and strengthening identity and self-efficacy.
Hello Sunday Morning (HSM)	Carah et al (2017) [2]; Black et al (2020) [28]; Kirkman et al (2018) [30]	Core principles of HSMs behavioral change model are mindfulness and community, to promote reflection on the user’s relationship with alcohol.
r∕StopDrinking	Colditz et al (2023) [32]	Social support: emotional, appraisal, informational, instrumental
In The Rooms	Bergman et al (2017) [23]	Recovery through exposure to relatable role models, enhancing motivation and strengthening identity and self-efficacy. Most resources were grounded in 12-step mutual help philosophy.
Soberistas	Sinclair et al (2017) [12]; Chambers et al (2017) [31]	Web-based mutual aid that is nonprescriptive, nonreligious, and nonjudgmental.
Unnamed	Liang et al (2021) [25]	Self-management support for opioid use during pregnancy.
Unnamed	Mudry and Strong (2013) [27]	Community of practice.

### What Are the Reported Outcomes of Web-Based Peer-Supported Forums?

In 4 of the 14 studies, authors used quantitative methods to report on outcomes related to the use of web-based peer-supported forums. The outcomes reported related to participation, perceived benefits of participation, and alcohol consumption.

Colditz et al [32] used mixed methods to characterize the social support provided on a StopDrinking recovery forum hosted on Reddit. Qualitative content analysis of 1386 responses to posts was undertaken to identify the type of social support provided: emotional, appraisal, or informational. The linguistic characteristics of these responses were quantified based on text length, complexity, and sentiment variables. Emotional support was coded as most common, and these responses were significantly shorter, less complex, and more positive than responses without emotional support, indicating that this type of response was a quick and easy way to exchange support among participants who could benefit from brief encouragement.

Bergman et al [23] examined the participation in ITR, primarily for people in, or working toward recovery, with a focus on abstinence, and surveyed 123 ITR users to examine their participation and perceived benefits of participation [23]. Participation was measured using ordinal scales to assess past-90-day ITR log-in frequency and intensity. The ITR users also reported their level of agreement with four statements on perceived benefit from participation: (1) enhanced recovery motivation, (2) enhanced recovery self-efficacy, (3) reduced craving, and (4) strengthened recovery identity. The ITR users engaged on average 30 minutes per day several times each week. Engagement was generally endorsed as helpful, particularly with respect to increased abstinence or recovery motivation and self-efficacy. Compared with ITR users who reported being abstinent for at least 1 year, those abstinent for less than 1 year showed similar rates of engagement with activities and similar levels of perceived benefit.

Schwebel and Orban [22] extended the study by Bergman et al [23] by examining the participation in a private, forum-based support group for changing alcohol use (Harm Reduction, Abstinence, and Moderation Support; HAMS), while not with an abstinence focus. For this study, 343 HAMS users were surveyed to examine their participation and perceived benefits of participation, measured through a modified version of Bergman et al [23] scales for participation. Perceived benefit was measured on a 5-point response scale assessing changing substance use behavior, craving, substance use behavior change self-efficacy, and substance use change motivation. The forum users most frequently reported visiting HAMS via Facebook daily, and up to 30 minutes per day. Most users somewhat or strongly agreed that HAMS helped them feel better about changing use, increased motivation for changing use, and increased self-efficacy for reaching or maintaining their substance use goals.

Kirkman et al [30] reported on the registration data of 1917 HSM users who signed up for 3 months of abstinence. To determine whether alcohol consumption changes were associated with participation, users completed the Alcohol Use Disorders Identification Test [34] at baseline and completion of the 3-month period. The HSM users who reported hazardous and harmful consumption levels, and engaged in the program, reported a significant decrease in alcohol consumption, achieving low-risk consumption levels 4 months after starting the program. Those who reported high-risk or dependent consumption levels before HSM engagement experienced the biggest reduction. These reductions in risk were maintained by forum users 7 months after starting HSM.

## Discussion

### Principal Findings

We identified 14 primary studies reporting on the use of web-based peer support for people experiencing addiction, for a variety of substances and behaviors, published between 2013 and 2023. While not all studies reported demographic data, of the 9 that did, the majority of web-based forum users were reported as middle-aged women participating in forums focusing on alcohol use. Participation in web-based peer-supported forums was reported both quantitatively (eg, number of forum users, length of time per visit, number of posts) and qualitatively (eg, information sharing, seeking support, sharing experiences). The web-based peer-supported forums were reportedly underpinned by a range of key principles, mostly mutual help approaches and recovery identity formation. Only 4 included studies reported on outcomes for forum users; however, these studies were observational: it is not possible to draw conclusions about the impact of participation in forums.

Web-based peer support can help to overcome barriers to attending traditional, face-to-face forums such as 12-step meetings, by enabling people experiencing addiction improved access to seek support beyond their physical location and with the benefit of anonymity [9]. For example, for people in regional and rural locations, web-based services such as web-based counseling are increasingly being used for people who have difficulty engaging with or accessing face-to-face services [38]. These benefits may extend to web-based peer-supported forums for people experiencing addiction, as demonstrated recently in an Australian mental health web-based forum, where participants described the importance of connection through peer support [39]. Further research in the field of addiction is warranted, particularly for people who are geographically isolated.

Although two-thirds of clients receiving alcohol and other drug treatment in Australia are men [40], our finding that women (particularly those aged between 40 years and early 50 years) were disproportionately represented among forum users is consistent with broader gender trends in help-seeking and life responsibilities. Women are more likely to seek health information, and are more likely to do so via the web, compared with men [41-44]. Women also experience a range of factors that restrict their access to formal addiction support [45,46], including caring responsibilities and social stigma [47]. Our finding adds to a growing body of literature that suggests nontraditional alcohol and other drug digital services (such as telehealth, telephone-based, and web-based interventions) may be filling a service gap for women [48,49]. Women’s overrepresentation may indicate that, due to the reduced cost and increased availability and accessibility of web-based peer-supported forums, receiving web-based addiction support may be more feasible than accessing traditional, offline services.

We found inconsistent reporting of web-based peer-supported forum participation and use. Forums were regularly reported as a source of information and support for people in similar circumstances. Information sharing was reported as useful for all forum users, even “passive” ones (“lurkers”). However, there was limited information distinguishing between the different ways users engaged with the forum. This nuance has been captured by other studies investigating nonaddiction forums. For example, in a recent publication on the role of group dynamics in shaping social support through web-based health communities, James et al [50] presented a model that focuses on active and passive use within such communities [50]. In their model, information sharing was a measure of active use, and information consumption was a measure of passive use. James et al [50] hypothesized that these 2 activities encompassed what people “do” in communities such as web-based forums [50]. Their emerging “web-based health community social support model” outlines how information consumption and sharing predict received social support through the forum. For administrators of web-based peer-supported forums, measuring the frequency of information consumption (eg, reading posts) and information sharing may be more useful than the number of forum users, posts, threads, and “likes.”

There was a mix of key principles underpinning the web-based peer-supported forums included in this review. The most frequent approaches reported were behavioral change, social identity, and mutual aid. The Social Identity Model of Recovery describes recovery as a process of change in a person’s social identity from being defined by membership of a group of people whose norms and values center on substance misuse, to membership of a group of people whose norms and values encourage recovery [35]. This socially embedded process is reflected in the included studies, particularly through the peer support element of web-based forums. The study included in this review by Colditz et al [38] highlighted the importance of emotional support to participants in an abstinence-based drinking forum, whereby although emotional support was provided in short text responses, even brief expressions of encouragement were valuable. Seeking peer support on the web, through sharing experiences and knowledge, for example, is gaining traction among people experiencing addiction [6]. This concept of sharing, particularly with people who have lived or living experience of addiction is a key principle of recovery [17]. Further research incorporating the Social Identity Model of Recovery within web-based peer-supported forums would assist in understanding how this socially embedded process occurs.

In line with other reviews of web-based support for people experiencing addiction, evidence on the benefits and effectiveness of web-based peer-supported forums remains lacking [18,51]. Face-to-face peer support remains a more evidence-based approach [52]. Recent guidelines to qualitatively analyze web-based support forums provide practical and methodological issues to consider when undertaking forum research [53]. However, there remain conceptual, theoretical, and methodological considerations, such as a lack of clarity around definitions of web-based forums (or communities), the dimensions of participation, and the need for experimental designs [18,53]. For example, questions remain in relation to when people engage with web-based peer-supported forums, for how long, and how web-based forums are positioned within and outside the treatment system. Understanding what works, for whom, how, and in what web-based contexts, requires further investigation.

### Limitations

Our scoping review has some limitations. First, our analysis is limited to those web-based peer-supported forums described in the studies identified via our search strategy. This means that some web-based forums may be overrepresented (eg, HSM, Soberistas, and alcohol-focused forums), while other forums may not be reported in the literature. Our findings were also limited to papers available in English, with our resulting sample featuring forums from only 5 countries, of which the majority (4/5) were Western, high-income, and English-speaking countries (Australia, Canada, the United Kingdom, and the United States). It is, therefore, unclear whether these findings are generalizable to other countries and cultures. In addition, text in a study title or abstract may not have referred to our specific search terms, and therefore, may have been missed. Since our research questions were based on peer-reviewed literature, we did not include gray literature or conference proceedings. Additionally, following Arksey and O’Malley’s [20] methodology for scoping reviews, we did not include quality appraisals as well. Papers from the computer science field are likely to be underrepresented. Due to the diverse and limited literature on this topic, we did not attempt to restrict our search to research on the effectiveness of web-based peer-supported forums. Instead, using the strengths of the scoping review approach, we brought together a heterogeneous body of literature that included descriptions and participation in web-based forums, as well as changes in substance use and other measures of effectiveness.

### Conclusions

Web-based peer-supported forums are used by people experiencing addiction in a number of ways, to share information and experiences, and give and receive support. Seeking web-based support offers an alternative approach to traditional face-to-face support options, and may reduce some barriers to engaging in peer support. Further research will assist forum users and forum administrators to articulate and optimize the benefits of web-based forum participation.

## Appendix

Multimedia Appendix 1 Search string for MEDLINE.

Multimedia Appendix 2 PRISMA-ScR Checklist.

## Abbreviations

D-RSS: digital recovery support service
HAMS: Harm reduction, Abstinence, and Moderation Support
HSM: Hello Sunday Morning
ITR: InTheRooms
