# Supplementary material for: Web-Based Forums for People Experiencing Substance Use or Gambling Disorders: Scoping Review
Source: JMIR Ment Health. 2024 Jun 17;11:e49010. doi: 10.2196/49010 (PMC11217707; doi:10.2196/49010)
Supplement: Multimedia Appendix 1 [file mental_v11i1e49010_app1.docx]

Box S1: Search string for Medline

1. Behavior, Addictive/

2. (addicti* adj1 behavio?r*).ti,ab.

3. Substance-Related Disorders/

4. exp Substance-Related Disorders/

5. ((substance* or drug* or alcohol* or prescription* or opioid* or gambl*) adj1 (abuse* or use* or dependenc* or addict* or disorder*)).ti,ab.

6. Gambling/

7. Online Social Networking/

8. ((on?line or internet or web*) adj2 (communit* or social* or forum* or interven* or network* or media or communicat* or counsel?ing or counsel?or* or self?help or self-help or peer* or support*)).ti,ab.

9. (outcome* or characteris* or experience* or model* or evaluat*).ti,ab.

10. ((psycholog* or psychosocial* or social*) adj1 (support* or assist* or encourag* or engag* or connect*)).ti,ab.

11. 1 or 2 or 3 or 4 or 5 or 6

12. 7 or 8

13. 9 and 10 and 11 and 12
